# The Effects of DPP-4 Inhibitors, GLP-1RAs, and SGLT-2/1 Inhibitors on Heart Failure Outcomes in Diabetic Patients With and Without Heart Failure History: Insights From CVOTs and Drug Mechanism

**DOI:** 10.3389/fendo.2020.599355

**Published:** 2020-12-01

**Authors:** Xiaohui Pan, Shishi Xu, Juan Li, Nanwei Tong

**Affiliations:** Department of Endocrinology and Metabolism, West China Hospital of Sichuan University, Chengdu, China

**Keywords:** type 2 diabetes, hypoglycemic agents, heart failure, cardiovascular benefit, cardiovascular outcome trials

## Abstract

Patients with type 2 diabetes (T2D) have a higher risk of heart failure (HF) than healthy people, and the prognosis of patients with diabetes and current or previous HF is worse than that of patients with only diabetes. We reviewed the HF outcomes in recently published cardiovascular outcome trials (CVOTs) of three new classes of anti-diabetic agents, namely, dipeptidyl peptidase-4 inhibitors (DPP-4is), glucagon-like-peptide 1 receptor agonists (GLP-1RAs), and sodium glucose cotransporter-2 inhibitors (SGLT-2is) or SGLT-2 and SGLT-1 dual inhibitors and divided the patients into two groups based on the history of HF (with or without) and analyzed their risks of HHF based on the receipt of the aforementioned anti-diabetes drug types. Since the follow-up period differed among the trials, we expressed the rate of HHF as events/1,000 person-years to describe the HF outcome. At last we pooled the data and analyzed their different effects and mechanisms on heart failure outcomes. Although DPP-4is did not increase the risk of HHF in T2D patients with a history of HF, they were associated with a significantly higher risk of HHF among patients without history of HF. Some GLP-1RAs reduced the risk of macrovascular events, but none of these drugs reduced the risk of HHF in patients with T2D irrespective of their HF history. It was not clarified whether SGLT-1/2is can improve the prognosis of macrovascular events in patients with T2D, but these drugs reduced the risk of HHF regardless of patients’ histories of HF. This information may be useful or referential for the “precise” selection of hyperglycemic medications. Further researches still needed to clarify the mechanisms of these anti-diabetic medications.

## Introduction

Cardiovascular disease (CVD) is a general concept describing several specific diseases, among which the atherosclerotic cardiovascular disease (ASCVD) and heart failure (HF) are major clinical concerns. Diabetes is an independent risk factor for both ASCVD and HF (1, 2). The Framingham Heart Study found that the risks of HF in male and female patients with type 2 diabetes (T2D) are 2.4- and 5-fold higher than that in their counterparts, respectively (2). Conversely, the presence of HF is an independent risk factor for diabetes. In total, 40% of patients with HF have been diagnosed with T2D (3). HF was once considered the frequent, forgotten, and often fatal complication of diabetes (4). The poor prognosis of patients with HF and diabetes has been linked to underlying diabetic cardiomyopathy exacerbated by hypertension and ischemic heart disease (5, 6). At present, HF is one of the most common CVDs in patients with diabetes (7).

Whereas diabetes increases the risk of CVD, anti-diabetic agents have some effects on the cardiovascular system that independent of glucose lowering. Cardiovascular outcome trials (CVOTs) of anti-diabetic agents are designed to clarify their cardiovascular safety in addition to their hypoglycemic effects (8). Along with the classic outcome of major adverse cardiovascular events (MACE, including cardiovascular death, myocardial infarction and stroke), recently published CVOTs of hypoglycemic drugs included analysis of HF data, especially the rate of hospitalization for heart failure (HHF). These results shed light on the direct or indirect effects of newly developed hypoglycemic agents on HF outcomes (HFOs), providing evidence for clinical decision-making and basic researches.

## Strategy and Analysis Methods

HF is a major cardiovascular condition with severe adverse consequences, including a substantially increased risk of death among patients with T2D (5). Thus, it is important to consider the history of HF when selecting treatments for patients with diabetes. But the effects and mechanisms of new anti-diabetic agents on HFO in patients with T2D with and without HF have not been discussed in detail and systematically reviewed previously. We reviewed the published CVOTs and HFOs in two subgroups (with or without histories of HF) for three types of new anti-diabetic agents, namely dipeptidyl peptidase-4 inhibitors (DPP-4is), glucagon-like-peptide 1 receptor agonists (GLP-1RAs), and sodium-dependent glucose cotransporter-2 inhibitors (SGLT-2is) or SGLT-2 and SGLT-1 inhibitors (SGLT-2/1is). Because of the limited access to the raw data and insufficient HF information in these studies, we selected the most commonly used heart failure endpoint HHF as the HFO of our analysis.

We searched the PubMed, Embase, and Ovid database for CVOTs and relevant subgroup analyses published through March 2020. The search terms were as follows: type 2 diabetes mellitus; hypoglycemic agents; dipeptidyl peptidase-4 inhibitor, glucagon-like-peptide 1 receptor agonist, sodium and sodium glucose cotransporter-1/2 inhibitor, and HF. Because we mainly used the results of CVOTs, any published CVOTs of the aforementioned three classes of hypoglycemic agents and subgroup analyses using the same data or population including HHF as an outcome were eligible for this analysis (see **Table 1**). We collected data from eligible trials using a self-designed data extraction form. The recorded study characteristics included the population, number of patients (in different subgroups, especially the history of HF), treatment protocol, median follow-up period, number of patients with a reported outcome for HFF, and the existing HFF rate.

**Table 1:** Characteristics of included cardiovascular outcome trials.

CVOTs Acronym (reference)	Agents(class/name)	Year of publication	No. of Participants	Participants with HF history (%)	Administration dose/rate	Follow-up time (median)
SAVOR-TIMI 53 (9)	DPP-4i/saxagliptin	2013	16,492	2,105(12.7)	2.5–5 mg PO/QD	2.1 years
EXAMINE (10)	DPP-4i/alogliptin	2013	5,380	1,533(28.5)	2.5–6.25 mg PO/QD	533 days
TECOS (11)	DPP-4i/sitagliptin	2015	14,671	2,643(18.0)	50–100 mg PO/QD	3.0 years
CARMELINA (12)	DPP-4i/linagliptin	2018	6,979	1,461(20.9)	5 mg PO/QD	2.2 years
EXILA (13)	Ex-4D/lixisentide	2015	6,080	1,358(22.4)	10–20 ug SC/QD	25 months
EXSCEL (14)	Ex-4D/exentide	2017	14,752	2,389(16.2)	2 mg SC/QW	3.2 years
LEADER (15)	hGLP-1A/liraglutide	2016	9,340	1,667(17.8)	1.8 mg SC/QD	3.8 years
SUSTAIN-6 (16)	hGLP-1A/semaglutide	2016	3,297	573(17.4)	0.5–1.0 mg SC/QW	2.1 years
Harmony (17)	hGLP-1A/albiglutide	2018	9,463	1,922(20.3)	30–50 mg SC/QW	1.6 years
REWIND (18)	hGLP-1A/dulaglutide	2019	9,901	853(8.6)	1.5 mg SC/QW	5.4 years
EMPA-REG (19)	SGLT-2i/empagliflozin	2015	7,020	706(10.1)	10–25 mg PO/QD	3.1 years
CANVAS (20)	SGLT-2i/canagliflozin	2017	10,142	1,461(14.4)	100–300 mg PO/QD	126.1 weeks
DECLARE (21)	SGLT-2/1i/dapagliflozin	2019	17,160	1,724(10.0)	10 mg PO/QD	4.2 years

Trials are listed by class of agents and starting calendar year (older to newer). CVOT indicates cardiovascular outcome trial; HF, heart failure; DPP-4i, dipeptidyl peptidase-4 inhibitor; Ex-4D, extendin-4 derivatives; hGLP-1A, human glucagon-like-peptide 1 analog; SGLT-2i, sodium glucose cotransporter-2 inhibitor; SGLT-1/2i, sodium glucose cotransporter-2 and -1 dual inhibitor; SAVOR TIMI 53, The Saxagliptin Assessment of Vascular Outcomes Recorded in Patients with Diabetes Mellitus (SAVOR)–Thrombolysis in Myocardial Infarction (TIMI) 53 trial; EXAMINE, Examination of Cardiovascular Outcomes with Alogliptin versus Standard of Care; TECOS, The Effect on Cardiovascular Outcomes in Type 2 Diabetes of Sitagliptin; CARMELINA, The Cardiovascular and Renal Microvascular Outcome Study with Linagliptin; ELIXA, The Evaluation of Lixisenatide in Acute Coronary Syndrome; EXSCEL, Exenatide Study of Cardiovascular Event Lowering; EXSCEL, Exenatide Study of Cardiovascular Event Lowering; LEADER, Liraglutide Effect and Action in Diabetes: Evaluation of Cardiovascular Outcome Results; SUSTAIN-6, Trial to Evaluate Cardiovascular and Other Long-term Outcomes with Semaglutide in Subjects with Type 2 Diabetes; Harmony, Albiglutide and cardiovascular outcomes in patients with type 2 diabetes and cardiovascular disease; REWIND, Dulaglutide and cardiovascular outcomes in type 2 diabetes; EMPA-REG OUTCOME, The Empagliflozin Cardiovascular Outcome Event Trial in Type 2 Diabetes Mellitus Patients; CANVAS, The Canagliflozin Cardiovascular Assessment Study; DECLARE, The Dapagliflozin Effect on Cardiovascular Events; PO, oral administration; SC, subcutaneous injection; QD, daily; QW, weekly.

Considering the different follow-up times of the studies, it was inappropriate to compare the outcomes of the randomized control trials (RCTs) using the number of HHF events alone, and thus, we collected the median follow-up time and number of events (HHF) and integrated these data to express the HHF rate as events/1,000 person-years. In brief, the number of patients with an HHF event in the trials was divided by the median follow-up time (in years) and the total number of patients in the treatment or placebo group (per 1,000 people). For the pooled analysis, we used Review Manager Software, Version 5.3 (Cochrane Collaboration, London, United Kingdom). The overall effect of the hypoglycemic agents on outcomes was calculated using the Mantel–Haenszel statistical method, and data were expressed as the hazard ratio (HR) and 95% confidence interval (CI). GraphPad Prism 7.00 (GraphPad Software, San Diego, CA, USA) was used to draw the forest plots.

### DPP-4is

DPP-4 is an enzyme expressed on the surface of most cell types that can cleave a broad range of substrates, such as glucagon like peptide-1 (GLP-1) and glucose-dependent insulinotropic polypeptide (GIP). DPP-4 inhibition can promote insulin secretion by islet β-cells and inhibit glucagon secretion by islet α-cells by prolonging the effects of GLP-1 and GIP *in vivo* (22), thereby reducing blood glucose levels. Although the risk of hypoglycemia is low, DPP-4is do not have cardiovascular benefits, and the relationship between DPP-4i use and HF risk has been a concern since their clinical application on.

**Figure 1:**
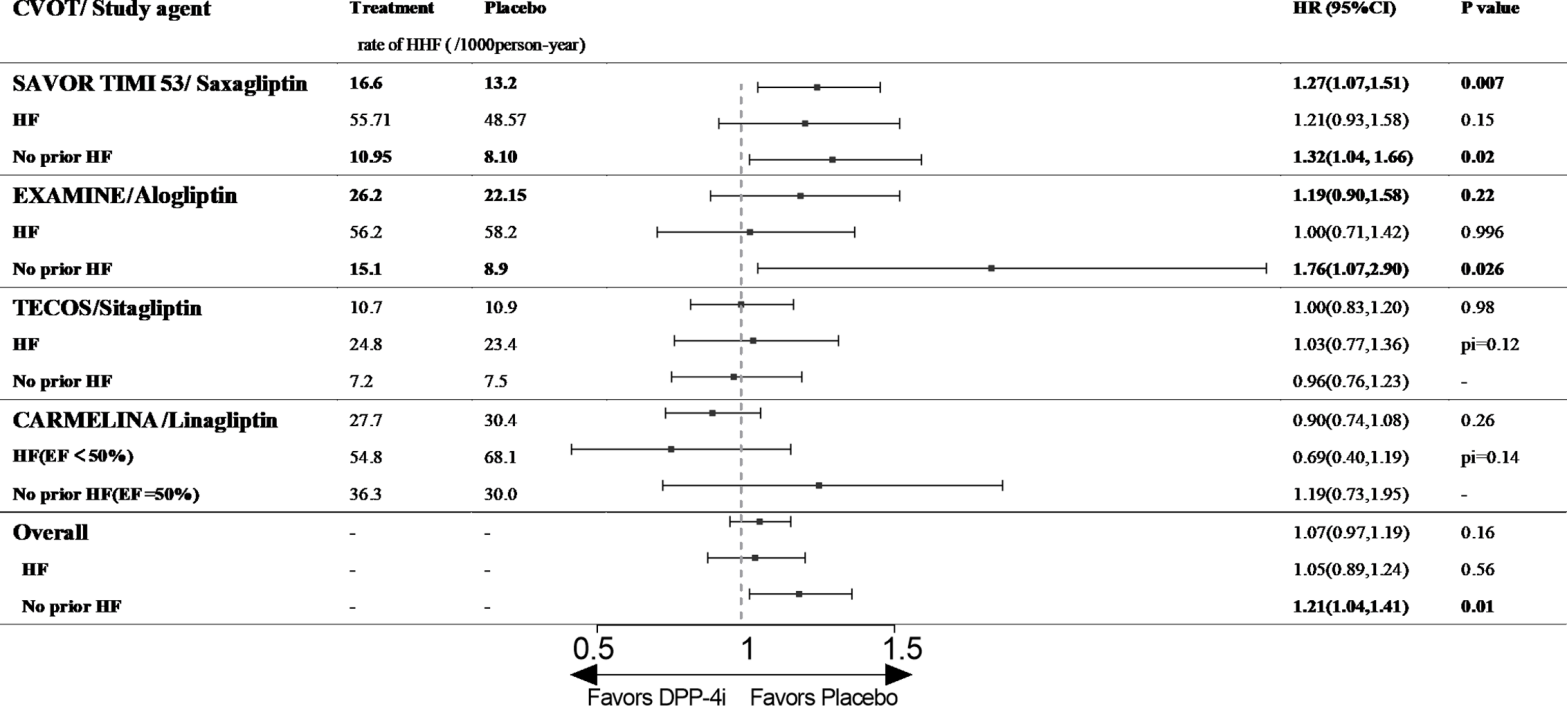
The effect of DPP-4is on HHF in type 2 diabetic patients with and without history of HF. CVOT, cardiovascular outcome trial; HHF, hospitalization for heart failure; HF, heart failure; EF, ejection fraction; pi, p value of interaction; SAVOR TIMI 53, The Saxagliptin Assessment of Vascular Outcomes Recorded in Patients with Diabetes Mellitus (SAVOR)–Thrombolysis in Myocardial Infarction (TIMI) 53 trial; EXAMINE, Examination of Cardiovascular Outcomes with Alogliptin versus Standard of Care; TECOS, The Effect on Cardiovascular Outcomes in Type 2 Diabetes of Sitagliptin; CARMELINA, The Cardiovascular and Renal Microvascular Outcome Study with Linagliptin.

Saxagliptin: SAVOR-TIMI 53**** (The Saxagliptin Assessment of Vascular Outcomes Recorded in Patients with Diabetes Mellitus [SAVOR]–Thrombolysis in Myocardial Infarction [TIMI] 53 trial) enrolled 16,492 patients with T2D (78.6% with established ASCVD), among whom 12.8% had a prior diagnosis of HF (New York Heart Association [NYHA] class II–III). The median follow-up time was 2.1 years (9). The HHF rates in the saxagliptin and control groups were 16.6 and 13.2/1,000 person-years, respectively (HR 1.27, 95% CI,1.07–1.51, p= 0.02), indicating that saxagliptin increased the risk of HHF in patients with T2D. When we grouped patients by history of HF, we found that saxagliptin did not increase the risk of HHF among patients with a previous history of HF (55.71/1,000 person-years vs. 48.57/1,000 person-years, HR 1.21, 95% CI 0.93–1.58, p= 0.15, **Figure 1**). However, among/ patients without HF history at baseline, saxagliptin significantly increased the risk of HHF (10.95/1,000 person-years vs. 8.10/1,000 person-years, HR 1.32, 95% CI 1.04–1.66, P = 0.02, **Figure 1**) (23).

Alogliptin: EXAMINE (Examination of Cardiovascular Outcomes with Alogliptin versus Standard of Care) enrolled 5,380 patients with T2D who were diagnosed with acute coronary syndrome (100% with established ASCVD) within 15–90 days before randomization, and 28% of these patients had a history of HF at baseline (before or after the index acute coronary syndrome event, recorded by the physician investigator). The median follow-up time in EXAMINE**** was 533 days. This study had the most patients with histories of HF among published CVOTs. In addition, the participants had a higher risk of cardiovascular events at baseline because they were diagnosed with acute coronary syndrome before enrolment (10). Although alogliptin did not increase the risk of HHF in the entire patient cohort (26.2/1,000 person-years vs. 26.0/1,000 person-years, HR 1.19, 95% CI 0.90–1.58, p= 0.2, **Figure 1**) or among patients with a previous history of HF (56.2/1,000 person-years vs. 58.2/1,000 person-years, HR 1.00, 95% CI 0.71–1.42, p= 0.996, **Figure 1**), the drug significantly increased the risk of HHF among patients with no history of HF (15.1/1,000 person-years vs. 8.9/1,000 person-years, HR 1.76, 95% CI 1.07–2.90, p= 0.026, **Figure 1**) (10, 24).

Sitagliptin: TECOS**** (The Effect on Cardiovascular Outcomes in Type 2 Diabetes of Sitagliptin) included 14,671 patients with T2D (100% with established ASCVD), 16.8% of whom had a history of HF (not defined, the NYHA class was provided for some participants). During a median follow-up of 3.0 years, sitagliptin did not increase the risk of HHF among the entire cohort (10.7/1,000 person-years vs. 10.9/1,000 person-years, HR 1.00, 95% CI 0.83–1.20, p= 0.98, **Figure 1**). Among patients with T2D and prior HF, sitagliptin did not increase the risk of HHF (24.8/1,000 person-years vs. 23.4/1,000 person-years, HR 1.03, 95% CI 0.77–1.36, p for interaction = 0.12, **Figure 1**), and similar results were obtained among patients with no prior HF history (30.7/1,000 person-years vs. 33.1/1,000 person-years, HR 0.96, 95% CI 0.76–1.23, p for interaction = 0.12, **Figure 1**). In a post hoc analysis, the risk of HHF among patients treated with sitagliptin did not differ by baseline HF severity (NYHA I or ≥II, NYHA class not reported) (11, 25).

Linagliptin: CARMELINA****** (The Cardiovascular and Renal Microvascular Outcome Study with Linagliptin) enrolled 6,979 patients with T2D and high CVD (100% with established ASCVD) and renal risks, and 26.8% of patients had a history of HF at baseline (not defined). The median follow-up time was 2.2 years (12). The risk of HHF did not differ between the linagliptin and placebo groups (27.7/1,000 person-years vs. 30.4/1,000 person-years, HR 0.90, 95% CI 0.74–1.08, p= 0.26, **Figure 1**). A post hoc analysis of CARMELINA****** used left ventricular ejection fraction (LVEF) as a criterion for analyzing the effect of linagliptin on cardiovascular events in patients with T2D. The results illustrated that linagliptin did not increase the risk of HHF among all patients or those with ejection fraction (EF) < 50% (54.8/1,000 person-years vs. 68.1/1,000 person-years, HR 0.69, 95% CI 0.40–1.19, p for interaction = 0.14, **Figure 1**). However, a numerical increase was observed in patients with EF >50% (36.3/1,000 person-years vs. 30.0/1,000 person-years, HR 1.19, 95% CI 0.73–1.95, p= 0.14, **Figure 1**) (26).

In conclusion, our pooled analysis illustrated that DPP-4is do not increase the risk of HHF among patients with T2D and a previous history of HF, but intriguingly, the drugs increase the risk of HFF among patients without history of HHF (because nearly all subjects had established ASCVD; HR 1.21, 95% CI 1.04–1.41, p= 0.01, **Figure 1**).

The inhibition of DPP-4 exerts diverse actions on heart, mainly mediated by its products stromal cell-derive factor-1(SDF-1) and GLP-1. SDF-1 is a stem cell chemokine that promoting inflammation, regeneration and repair (27). Inhibiting DPP-4 genetically or using DPP-4is can ameliorate SDF-1α–dependent microvasculopathy and diastolic left ventricular dysfunction associated with diabetes or overload pressure, improve cardiovascular outcomes after myocardial infarction in mice in animal models (not primates) of HF, possibly through a GLP-1/cyclic adenosine monophosphate (cAMP)-related mechanism (28–30). On the other hand, DPP-4 involves in the degradation of vasodilator factors and the nitric oxide-dependent mechanism, inhibition of DPP-4 can exert important systemic vasodilator effects that reduce heart load, despite attenuated in diabetes (31, 32). The release of SDF-1 by the heart recruits mesenchymal stem cells with regeneration potential, but the stem cells may be transformed into fibroblasts rather than cardiomyocytes in states of diabetes and other cardiac stress, thus exerts deleterious effects (33, 34). DPP-4i has positive inotropic effects by enhancing the actions of GLP-1 to stimulate cAMP in cardiomyocytes (35). Furthermore, SDF-1 augments the increase in myocardial cAMP promoted by the outflow of sympathetic activity from the central nervous system (36), elevated cAMP might lead to calcium overload and adverse effect on heart.

In one word, basic research suggested that DPP-4is could improve HFO, but these results were not replicated in human and exerted adverse effects in RCTs (37), which prompted the FDA to warn that DPP-4is should be used with caution in patients with T2D and a history of HF or kidney impairment (38). After we separately analyzed the HHF rate in patients with and without prior histories of HF, we found that DPP-4is did not increase the risk of HHF among patients with previousHF, whereas an elevated risk was identified among patients without previous HF. The mechanism underlying the divergent effects of DPP-4 inhibition on animal models and human RCTs is unclear. We did not find detailed HFO data in other primates treated with DPP-4is. It is known that DPP-4 is a broadly expressed protein with multiple functions, including roles in the immune system and cancer development in addition to glucose regulation. Moreover, the physiology and function of some DPP-4–like enzymes are unknown. Long-term DPP-4i use might alter immune homeostasis, but whether this effect is associated with their adverse effects on HF requires further investigation.

### GLP-1RAs

GLP-1 is an endogenous incretin that can lower blood glucose through multiple mechanisms, such as stimulating insulin synthesis, suppressing islet α-cell function, and promoting the proliferation and differentiation of β-cells, regulating gastric emptying and appetite while carries a low risk of hypoglycemia (39). Human GLP-1 has an extremely very short half-life *in vivo* because of the degradation by DPP-4 and renal elimination (40). The newly developed anti-diabetic agents, GLP-1RAs can resist degradation by DPP-4 while exerting the effects of GLP-1. GLP-1RAs can be divided into two types according to their structure, namely extendin-4 (Ex-4) derivatives and human GLP-1 (hGLP-1) analogs. Ex-4 derivates, including lixisenatide and exenatide, have 53% amino acid identity with hGLP-1. Liraglutide, semaglutide, albiglutide, and dulaglutide are modified active hGLP-1 analogs with more than 90% identity with hGLP-1 (41). Ex-4 derivatives and hGLP-1 analogs have different structures, receptors or different recognition sites on the same receptor, thus it is reasonable that they activate different signaling pathways and have different performance in RCTs (42, 43). CVOTs illustrated that Ex-4 derivatives did not improve the outcome of ASCVD, whereas hGLP-1 analogs displayed benefits against ASCVD, lowering the risk of MACE and/or single elements of MACE (cardiovascular death, myocardial infarction, and stroke), none of them improved the HFO (44).

**Figure 2:**
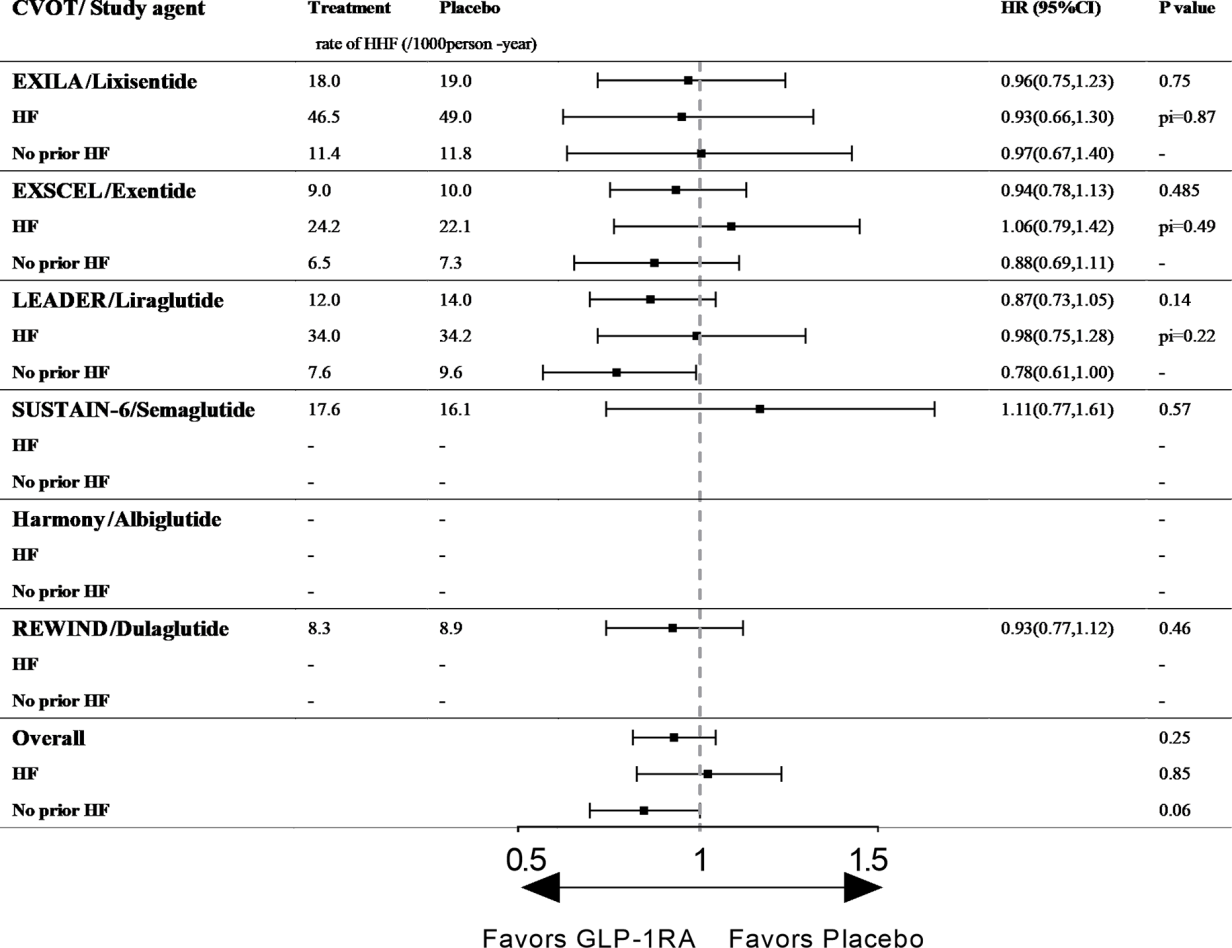
The effect of GLP-1RAs on HHF in type 2 diabetic patients with and without history of HF. CVOT, cardiovascular outcome trial; HHF, hospitalization for heart failure; HF, heart failure; Pi, p value of interaction; ELIXA, The Evaluation of Lixisenatide in Acute Coronary Syndrome; EXSCEL, Exenatide Study of Cardiovascular Event Lowering; LEADER, Liraglutide Effect and Action in Diabetes: Evaluation of Cardiovascular Outcome Results; SUSTAIN-6, Trial to Evaluate Cardiovascular and Other Long-term Outcomes with Semaglutide in Subjects with Type 2 Diabetes; Harmony, Albiglutide and cardiovascular outcomes in patients with type 2 diabetes and cardiovascular disease; REWIND, Dulaglutide and cardiovascular outcomes in type 2 diabetes.

Liraglutide: LEADER (Liraglutide Effect and Action in Diabetes: Evaluation of Cardiovascular Outcome Results) enrolled 9,340 patients with T2D (81% of whom had established ASCVD) and followed them for a median of 3.8 years. In total, 18% of patients had a history of HF (chronic HF [NYHA class I–III], left ventricular systolic or diastolic dysfunction on imaging) (15). Liraglutide is the first hGLP-1 analog with demonstrated cardioprotective effects and the only GLP-1RA that can reduce the risks of both MACE and CVD death. However, liraglutide did not alter the risk of HHF among the entire patient cohort (12.0/1,000 person-years vs. 14.0/1,000 person-years, HR 0.87, 95% CI 0.73–1.05, p= 0.14, **Figure 2**), and no effect was identified among patients with (34.0/1,000 person-years vs. 34.2/1,000 person-years, HR 0.98, 95% CI 0.75–1.28, p for interaction = 0.22, **Figure 2**) or without a history of HF (7.6/1,000 person-years vs. 9.6/1,000 person-years, HR 0.78, 95% CI 0.61–1.00, p for interaction = 0.22, **Figure 2**). However, liraglutide reduced the risk of the composite outcome of CVD or HHF among patients with T2D without a prior HF history (45, 46).

The effect of liraglutide on chronic HF (with or without diabetes) remains unclear. Some studies reported that liraglutide can improve LVEF (47, 48), whereas others reported a contradictory result. The recently published ***LIVE*** trial (Effect of liraglutide, a glucagon-like peptide-1 analogue, on left ventricular function in stable chronic HF patients with and without diabetes) was an RCT designed to observe the effect of liraglutide on heart function in patients with chronic HF (49). The study enrolled 241 patients with chronic HF and EF ≤ 45% (with and without T2D) and followed them for 24 weeks. The results of ***LIVE*** also indicated that liraglutide had no beneficial effects on LVEF. Moreover, the drug slightly increased the risk of adverse events because of an increased heart rate, which is common in clinical trials of GLP-1RAs (50). Thus, liraglutide has little benefit on the risk of HHF.

Ex-4 derivate lixisenatide: ELIXA (The Evaluation of Lixisenatide in Acute Coronary Syndrome trial) enrolled 6068 patients with T2D who had an acute coronary event (100% with established ASCVD) within 180 days before screening, and 22% of the patients had histories of HF (no defined criteria). The median follow-up period was 25 months in each study group (13). Among all participants, lixisenatide did not reduce the risk of HHF (18.0/1,000 person-years vs. 19.0/1,000 person-years, HR 0.96, 95% CI 0.75–1.23, p = 0.75, **Figure 2**). In the subgroup analysis by HF history, the effects of the drug did not differ between those with or without a history of HF (p for interaction = 0.87), and the therapy did not reduce the risk of HHF in either group (p= 0.75, **Figure 2**) (13).

Ex-4 derivate exenatide: EXSCEL (Exenatide Study of Cardiovascular Event Lowering) is the largest CVOT of GLP-1RAs to date. The study included 14,752 patients with T2D (73.1% with established ASCVD), and the median duration of follow-up was 3.2 years (14, 51). In total, 73.1% of the participants had established ASCVD, and 16% had histories of HF (the HF status at baseline was prospectively recorded by the clinician investigator based on all available clinical data including patients’ signs/symptoms and objective measures such as echocardiography and biomarker data [e.g., natriuretic peptide levels]). Exenatide did not increase the risk of HHF among patients with T2D with and without histories of HF (p=0.485 and p for interaction=0.33, respectively, **Figure 2**).

DPP-4is and GLP-1RAs are both incretin-based therapies that potentiates endogenous GLP-1, but their effects on the cardiovascular system vary a lot (52). As we mentioned previously, DPP-4is exerted some glucose-independent effects through a GLP-1/cAMP-mediated mechanism in animal models (mainly in rats or mice and not primates), part of these effects are cardioprotective. Unlike DPP-4i, the GLP-1RAs do not potentiate SDF-1 neither the subsequent effects on cardiovascular system (52). GLP-1RAs are directly cardioprotective both *in vitro* and *in vivo*, both in GLP-1R-dependent and independent pathways. The cardioprotective actions of native GLP-1 demonstrated in human and animal studies, perfused ischemic hearts and cardiomyocyte cultures are associated with GLP-1R-dependent induction of a cardioprotective gene and protein expression profile, which have been well reviewed elsewhere (40, 53). The non-GLP-1R-dependent pathways including local and systemic inflammation reduction and gut microbiome alteration (54–56), which make the specific cardioprotective mechanism of GLP-1RAs remain challenging.

GLP-1RAs can ameliorate endothelial dysfunction, improve myocardial function, and protect cardiomyocytes against glucolipotoxicity and ROS in animal models and humans (albeit with different effects) (57, 58). But the available evidence from RCTs, including CVOTs, are not sufficient to confirm that GLP-1RAs are better options for patients with T2D when considering their HF benefits. More studies aim on the effects and mechanism of GLP-1RAs on heart failure are still needed, both clinical and basic.

### SGLT-2is or SGLT-2/1is

Inhibiting SGLT-2 and SGLT-1 decrease glucose reabsorption at the proximal tubules and intestine respectively thereby increasing the excretion of glucose in urine or feces (59, 60). The class of medication does not act on β-cells thus unable to induce insulin secretion directly, but these drugs can act on α-cells and stimulate the secretion of glucagon which promotes glucogenesis in the liver (61), making them unique diabetes drugs withlower risks of hypoglycemia. Clinical data illustrated the promising effects of SGLT-2is or SGLT-2/1is on weight, blood pressure, uric acid controland lipid profiles. four SGLT-2is or SGLT-2/1is have been approved for treating diabetes so far, three of them finished the CVOTs, including the specific SGLT-2 inhibitors empagliflozin and dapagliflozin and the SGLT-2/1i canagliflozin. In this review, we categorized them respectively collectively as SGLT-2is or SGLT-2/1is (**Table 1**).

**Figure 3:**
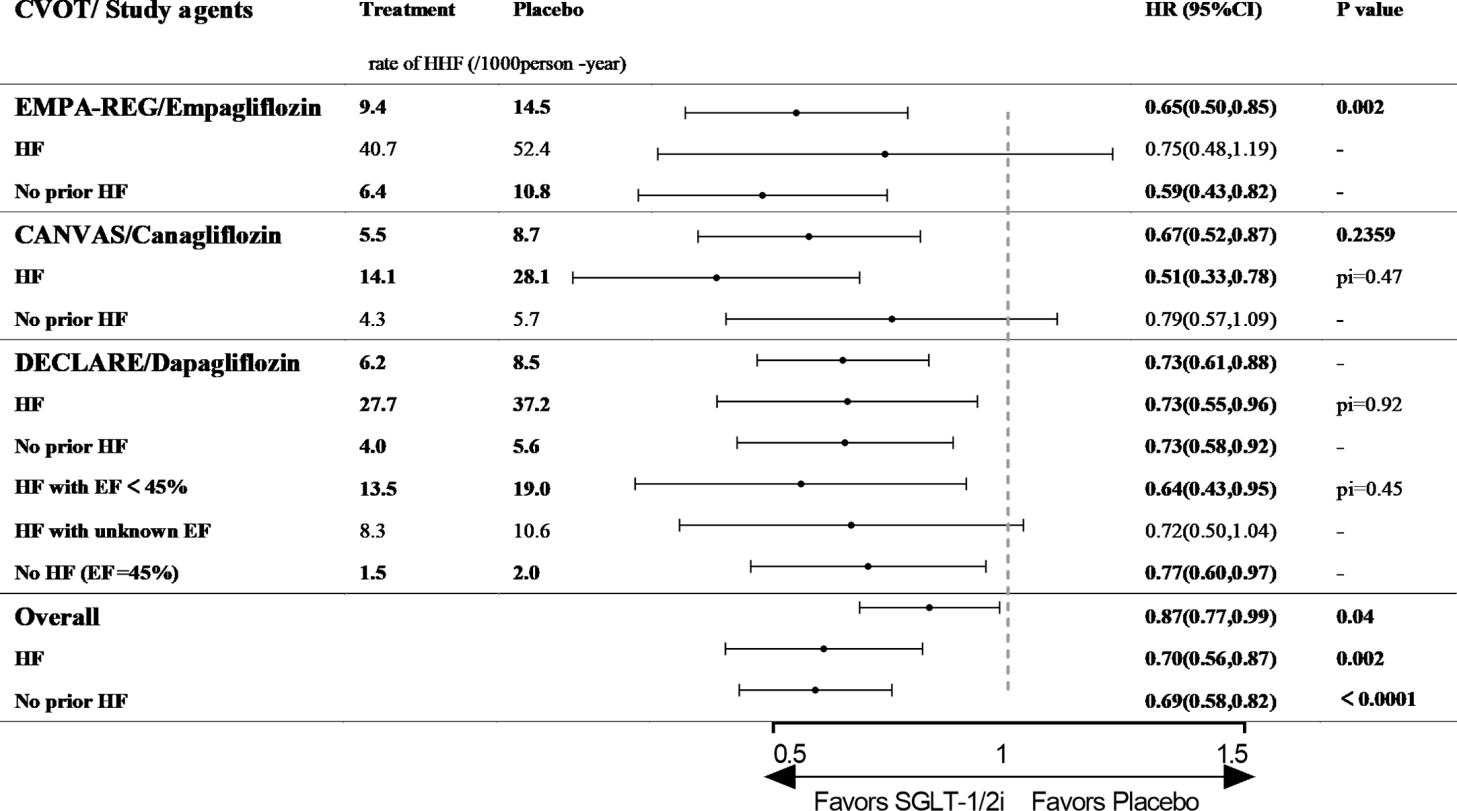
The effect of SGLT-2is or SGLT-2/1is on HHF in type 2 diabetic patients with and without history of HF. CVOT, cardiovascular outcome trial; HHF, hospitalization for heart failure; HF, heart failure; EF, ejection fraction; pi, p value of interaction; EMPA-REG OUTCOME, The Empagliflozin Cardiovascular Outcome Event Trial in Type 2 Diabetes Mellitus Patients; CANVAS, The Canagliflozin Cardiovascular Assessment Study; DECLARE, The Dapagliflozin Effect on Cardiovascular Events.

Empagliflozin: EMPA-REG OUTCOME (The Empagliflozin Cardiovascular Outcome Event Trial in Type 2 Diabetes Mellitus Patients) enrolled 7020 patients with T2D (100% with established ASCVD), 10% of whom had previous histories of HF (patients with investigator-reported HF at baseline were allowed to participate without any restriction regarding EF or NYHA class). The median follow-up duration was 3.1 years (19). The study revealed that empagliflozin lowered the risk of HHF by 35% among the overall cohort (9.4/1,000 person-years vs. 14.5/1,000 person-years, HR 0.65, 95% CI 0.50–0.85, p=0.002, **Figure 3**). Among patients with previous HF, the risk of HHF was numerically lower for empagliflozin than for placebo (40.7/1,000 person-years vs. 52.4/1,000 person-years, HR 0.75, 95% CI 0.48–1.19, p value is not available, **Figure 3**), although this result may be attributable to the small sample size of the analysis. However, the positive effect of empagliflozin on cardiovascular outcomes was significant among patients without prior history of HF (6.4/1,000 person-years vs. 10.8/1,000 person-years, HR 0.59, 95% CI 0.43–0.82, p value is not available, **Figure 3**) (62).

Canagliflozin: CANVAS (The Canagliflozin Cardiovascular Assessment Study) enrolled 10,142 patients with T2D (59.4% with established ASCVD), 14% of whom had previous histories of HF (based on a physician’s review of the patients’ medical histories at the first visit with no requirement for the measurement of diagnostic biomarkers or echocardiography). The median follow-up time was 2.4 years (20). Canagliflozin lowered the risk of HHF among the overall cohort (5.5/1,000 person-years vs. 8.7/1,000 person-years, HR 0.67, 95% CI 0.52–0.87, p value is not available, **Figure 3**). Among patients with previous HF, the risk of HHF was significantly lower in the canagliflozin arm (14.1/1,000 person-years vs. 28.1/1,000 person-years, HR 0.51, 95% CI 0.33–0.78, p for interaction= 0.47, **Figure 3**). Meanwhile, the risk of HHF was 21% lower for the canagliflozin arm among patients without previous HF (4.3/1,000 person-years vs. 5.7/1,000 person-years, HR 0.79, 95% CI 0.57–1.09, p for interaction= 0.47, **Figure 3**). Regarding the composite endpoint of CVD death or HHF, canagliflozin reduced the risk regardless of the prior history of HF (63).

Dapagliflozin: DECLARE (The Dapagliflozin Effect on Cardiovascular Events) is the largest and longest CVOT to date and dapagliflozin is the only SGLT-2/1i with a CVOT. The study included 17,160 participants (only 40.6% had not been diagnosed with CVD), and only 10% subjects had previous HF (no defined criteria). The patients were followed-up for a median of 4.2 years (21). Dapagliflozin reduced the risk of HHF by 27% among patients with T2D (6.2/1,000 person-years vs. 8.5/1,000 person-years, HR 0.73, 95% CI 0.61–0.88, p value is not available, **Figure 3**). When HF events were analyzed separately among patients with and without previous HF, dapagliflozin also significantly reduced the risk of HHF in both groups (p for interaction = 0.92, **Figure 3**) (64). In a post hoc analysis using EF of 45% as the threshold, dapagliflozin reduced the risk of HHF among patients with HF and EF<45% (13.5/1,000 person-years vs. 19.0/1,000 person-years, HR 0.64, 95% CI 0.43–0.95, p value is not available, **Figure 3**). Among patients without HF and EF>45%, the risk of HHF was reduced by 23% (1.5/1,000 person-years vs. 2.0/1,000 person-years, HR 0.77, 95% CI 0.60–0.97, p value is not available, **Figure 3**). Among patients with HF but an unknown EF, dapagliflozin did not alter the risk of HHF versus placebo (65).

In addition, dapagliflozin has been studied in patients with established HF. In the Dapagliflozin in Patients with Heart Failure and Reduced Ejection Fraction (***DAPD-HF***) study, 4,744 patients with HF (NYHA class II–IV) and EF ≤ 40% were enrolled and followed up for a median of 18.2 months (66). The primary outcome of the trial was a composite of HHF, an urgent visit resulting in intravenous therapy for HF, or death from cardiovascular causes. The risk of the primary outcome was reduced by 26% in the dapagliflozin group (107.1/1,000 person-years vs. 139.3/1,000 person-years, HR 0.74, 95% CI 0.65–0.85, p value is not available), whereas the risk was 25% lower among patients with HF and T2D (131.6/1,000 person-years vs. 167.6/1,000 person-years, HR 0.75, 95% CI 0.63–0.90, p value is not available). The results of ***DAPA-HF*** illustrated that dapagliflozin can reduce the risk of HHF among patients (EF≤40%) with chronic HF with and without T2D.

The benefits of SGLT-2is or SGLT-2/1is on cardiovascular system appeared early in the trials and persisted throughout the studies. The most significant effect of them on cardiovascular system relies on improving ventricular loading conditions. The osmotic and natriuretic diuresis reduces the preload of heart, combining with the afterload reduction induced by improvements on vascular function and blood pressure, the class of agent alleviates the fluid burden on ventricular effectively (21, 67). Decreased blood flow to the kidney and relative medullary hypoxia may increase erythropoietin production and increase red blood cell mass, which may improve cardiac oxygen delivery with higher hemoglobin levels (68). Except glucose control, the SGLT-2is or SGLT-2/1is increase the level of ketone body β-hydroxybutyrate (βOHB) moderately to provide fuel to the myocardium under stress and improve the metabolism of heart, which was called the thrifty substrate hypothesis (69–71). Moreover, SGLT-2is or SGLT-2/1is can alleviate heart failure by reducing cytoplasmic sodium and calcium levels through inhibition of Na^+^/H^+^ exchanger (NHE) 1 in the myocardium and/or NHE3 in proximal tubule (72, 73). As for the cardiac structure remodeling during heart failure, SGLT-2is or SGLT-2/1is suppress collagen synthesis *via* increasing the activation of M2 macrophages and inhibiting myofibroblast differentiation, attenuates TGF-β1-induced fibroblast activation and suppress expression of key pro-fibrotic markers, including type I collagen, α-smooth muscle actin, connective tissue growth factor and matrix metalloproteinase 2, thus prevents or alleviate myocardial fibrosis (74, 75), inhibit histone deacetylase and prevent pro-hypertrophic transcription pathways (76).

SGLT-2is or SGLT-2/1is is the first class of diabetes drugs to display a benefit for HFO, and all three SGLT-2is or SGLT-2/1is with CVOTs reduced the risk of HHF among patients with T2D regardless of their prior history of HF (P<0.001, **Figure 3**) (19, 20, 77, 78). Based on the strong evidence in the RCTs, SGLT-2is or SGLT-2/1is have been given a leading role in the treatment of HF in patients with T2D, and been recommended by significant guidelines (79–81). In the Standards of Medical Care in Diabetes-2020 released by the American Diabetes Association, SGLT-2is with an HF benefit have been recommended as add-on therapies for metformin in patients with T2D and HF, especially those with EF<45% (79).

## Conclusion

**Figure 4:**
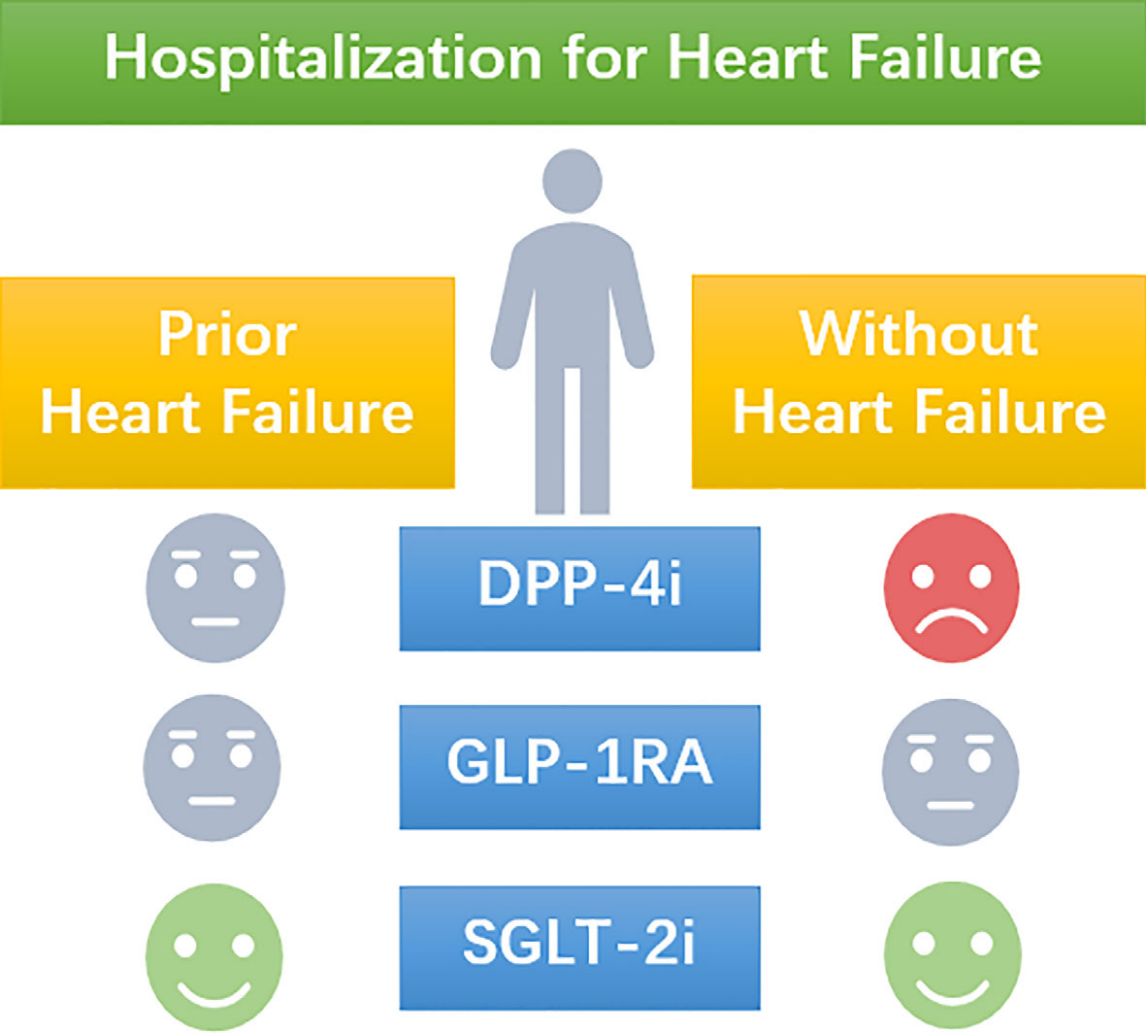
A schematic picture of the manuscript. The use of DPP-4i increases the risk of hospitalization for heart failure in T2D patients without prior heart failure, but present a neutral effect in T2D patients with prior heart failure. The use of GLP-1RA presents a neutral effect on the risk of hospitalization for heart failure no matter the heart failure status of T2D patients. SGLT-2i reduces the risk of hospitalization for heart failure no matter the heart failure status of T2D patients.

After comparing the HHF outcome of the three classes of medications in CVOTs using the unified unit events/1,000 person-years we found that the DPP-4is saxagliptin and alogliptin increased the risk of HHF among T2D patients without a history of HF. The sitagliptin and linagliptin did not increase nor decrease the risk of HHF in T2D patients no matter their heart failure condition. The GLP-1RAs, including hGLP-1 analogs and Ex-4 derives, had neutral effect on HHF regardless the history of HF, but more data on HFO of semaglutide, albiglutide, and dulaglutide are still needed to fulfill the comparison. It is not surprised to find that SGLT-2is can improve the HHF outcome in overall T2D patients. However, the benefit of empagliflozin covers the patients without prior HF while the canagliflozin covers those with prior HF, only the dapagliflozin benefits T2D patients both with and without prior HF on HHF (**Figure 4**). The mechanisms behind of the different effects of theses medication still needed further study. Based on the existing evidence, it is necessary to take the history of heart failure into consideration when prescript hypoglycemic medication to T2D patients.

## Discussion

The etiology of heart failure includes both micro- and macrovascular pathologies along with nerves and myocardium lesions, of which lots of risk factors contributing to its development and progression (82, 83). The complicated etiology and comorbidities make the treatment of heart failure a tough problem (80, 81). In T2D patients, the excessive risk of heart failure along with the elusive mechanisms of anti-hyperglycemic drugs on the cardiovascular system have brought more difficulties to the management of heart failure (6). Except the adverse effect of glucose dysregulation, the use of insulin and other oral anti-hyperglycemic drugs like TZDs have been associated with increased heart failure risk (84). The management of heart failure in T2D patients has raised more and more concerns recently. But the criterions of diagnosis and severity assessment of heart failure are complicated.

The history of HF plays an important role in the treatment and prognosis of both T2D and heart failure. The three recently introduced classes of antidiabetic medications, DPP-4is, GLP-1RAs and SGLT-2is or SGLT-2/1is, tent to exert different effects in T2D patients with different heart failure condition. Although mortality is the best criterion for evaluating HFOs, HHF has been considered a comprehensive criterion in recently published trials. Based on the current data, our review used the history of HF to divide patients with T2D into two groups (with or without prior HF) and analyzed the effect of different anti-diabetic agents on HFO (HHF). As a result, it is clear that no DDP-4i has cardiovascular benefits in patients with T2D. The reason and mechanism underlying the increased risk of HHF for DDP-4is among patients T2D and no previous history of HF is complicated and elusive, but the present data are sufficient to illustrate the need for caution when prescribing these drugs to patients with T2D. The hGLP-1 analog liraglutide reduced the risk of MACE, but it did not improve the prognosis of HF regardless of the prior HF history. The Ex-4 derivatives lixisenatide and exenatide had no cardiovascular benefits, including no effects on HF. According to the present evidence, we can only postulate that hGLP-1 analogs and Ex-4 derivatives do not increase or reduce the risk of HHF among patients with T2D. The SGLT-2i empagliflozin has more benefits in patients without a history of HF than in those with prior HF. Canagliflozin was primarily beneficial in patients with prior HF. Dapagliflozin can reduce the risk of HHF irrespective of the history of HF. In general, SGLT-2is or SGLT-2/1is can reduce the risk of HHF regardless of the history of HF, and they can be used in the prevention and treatment of HF in patients with T2D. The potential cardioprotective effects of SGLT-2is or SGLT-2/1is are of great promise and deserve deeper researches, the ongoing trials of the class of agent focus on heart failure patients with/without T2D will show us more hope for heart failure treatment.

Our study has several limitations. First, the classification of heart failure status in our analysis was based on the established subgroups information from the CVOTs. It is hard to conclude a criterion for the history of heart failure due to the definitions of heart failure are not unified in the CVOTs. In most trials, the heart failure status was recorded by the clinician-investigator based on clinical data, some trials did not give any information about the assessment HF. Fortunately, at least three agents in each class provided enough data on heart failure history and most of them are the same, that is “recorded by investigators”. Secondary, we extracted the data only from CVOTs rather than more RCTs with heart failure events, which might make our conclusion incomplete. The CVOTs have strict inclusion criterions, predesigned cardiovascular endpoints and longer follow-up periods. As the purpose of CVOTs is to detect the cardiovascular safety of anti-diabetic agents, the data collection would be focus on the cardiovascular effects, which fits our object better. However, as mentioned before, even CVOTs had some data on heart failure missed or unpublished. Like the GLP-1RAs semaglutide (16), albiglutide (17), and dulaglutide (18), which might obscure the true effect of their class on HFO. We believe our preliminary conclusion can aid the clinical management of patients with T2D and provide some clues for future studies. Further well-designed basic and clinical studies with comprehensive evaluation and assessment of heart failure are still needed to cover more aspects of this issue.

## Author Contributions

XP, SX, JL, and NT wrote the review article. All authors contributed to the article and approved the submitted version.

## Conflict of Interest

The authors declare that the research was conducted in the absence of any commercial or financial relationships that could be construed as a potential conflict of interest.

## Abbreviations

ASCVD, atherosclerotic cardiovascular disease; βOHB, β-hydroxybutyrate; cAMP, cyclic adenosine monophosphate; CANVAS, The Canagliflozin Cardiovascular Assessment Study; CARMELINA, The Cardiovascular and Renal Microvascular Outcome Study with Linagliptin; CVD, cardiovascular disease; CVOT, cardiovascular outcome trial; DAPD-HF, Dapagliflozin in Patients with Heart Failure and Reduced Ejection Fraction; DECLARE, The Dapagliflozin Effect on Cardiovascular Events; DPP-4i, dipeptidyl peptidase-4 inhibitor; Ex-4, extendin-4; EF, ejection fraction; ELIXA, The Evaluation of Lixisenatide in Acute Coronary Syndrome; EMPA-REG OUTCOME, The Empagliflozin Cardiovascular Outcome Event Trial in Type 2 Diabetes Mellitus Patients; EXAMINE, Examination of Cardiovascular Outcomes with Alogliptin versus Standard of Care; EXSCEL, Exenatide Study of Cardiovascular Event Lowering; GIP, glucose-dependent insulinotropic polypeptide GLP-1, glucagon like peptide-1; GLP-1RA, Glucagon-like-peptide 1 receptor agonist; HF, heart failure; HFO, heart failure outcome; HGLP-1A, human GLP-1 analogues; HHF, hospitalization for heart failure; LEADER, Liraglutide Effect and Action in Diabetes, Evaluation of Cardiovascular Outcome Results; LIVE, Effect of liraglutide, a glucagon-like peptide-1 analogue, on left ventricular function in stable chronic HF patients with and without diabetes; LVEF, left ventricular ejection fraction; MACE, major adverse cardiovascular events; NHE, Na^+^/H^+^ exchanger; NYHA, New York Heart Association; RCT, randomized controlled trials; SAVOR-TIMI 53, The Saxagliptin Assessment of Vascular Outcomes Recorded in Patients with Diabetes Mellitus (SAVOR)–Thrombolysis in Myocardial Infarction (TIMI) 53 trial; SDF-1, stromal cell-derive factor-1; SGLT-2is, sodium-dependent glucose cotransporter-2 inhibitor; SGLT-2/1i, sodium-dependent glucose cotransporter-2 and 1 dual inhibitor; T2D, type 2 diabetes; TECOS, The Effect on Cardiovascular Outcomes in Type 2 Diabetes of Sitagliptin.
